# Growth Characteristics of Sheep-Derived *Bacteroides fragilis* and Preliminary Research on Effects in Mice and Lambs

**DOI:** 10.3390/microorganisms13010087

**Published:** 2025-01-04

**Authors:** Cheng Cheng, Jinye Du, Jianping Tao, Darong Cheng

**Affiliations:** 1 College of Veterinary Medicine, Yangzhou University, Yangzhou 225009, China; 2 Jiangsu Co-Innovation Center for Prevention and Control of Important Animal Infectious Diseases and Zoonoses, Yangzhou 225009, China

**Keywords:** *Bacteroides fragilis*, probiotics, diarrhea, intestinal injury, intestinal microbiota

## Abstract

With the growing demand for sheep, the sheep farming industry has developed rapidly. However, lamb diarrhea, a disease with high mortality rates, significantly hampers the industry’s growth. Traditional antibiotic treatments often disrupt the Intestinal microbiota, induce antibiotic resistance, and cause adverse side effects, highlighting the urgent need to develop alternative therapies. *Bacteroides fragilis*, a candidate next-generation probiotic, has been closely associated with intestinal health. This study investigated the growth characteristics and probiotic effects of a sheep-derived *Bacteroides fragilis* isolate, focusing on its efficacy in alleviating lamb diarrhea and infectious intestinal diseases. The experiments demonstrated that the *Bacteroides fragilis* isolate grows well under mildly acidic conditions (pH 6–8), exhibits some tolerance to bile salts, and has survival rates of 38.89% and 92.22% in simulated gastric and intestinal fluids, respectively, indicating its potential as a probiotic. In a mouse model, *Bacteroides fragilis* intervention significantly alleviated colonic inflammation caused by *Enterohemorrhagic Escherichia coli* infection, enhanced tight junction protein expression, mitigated oxidative stress, and improved intestinal barrier function, with high-dose interventions showing superior effects. In lamb trials, *Bacteroides fragilis* intervention stopped diarrhea in four out of five lambs, partially restored intestinal microbiota diversity, and reduced the abundance of potential pathogens such as *Aerococcus suis* and *Corynebacterium camporealensis*. Therefore, *Bacteroides fragilis* exhibited remarkable effects in regulating intestinal homeostasis, alleviating inflammation, and promoting recovery from diarrhea.

## 1. Introduction

In animal husbandry, lamb diarrhea is a disease with both high incidence and mortality rates, especially in large-scale farming, which slows down production, resulting in a decrease in economic benefits [1]. Lamb diarrhea usually occurs in the early stages after birth and is characterized by varying degrees of diarrhea, dehydration, and depression. Depending on the cause, lamb diarrhea can be classified into several types, including seasonal diarrhea, bacterial diarrhea, viral diarrhea, parasitic diarrhea, toxic diarrhea, and dyspeptic diarrhea. Among them, bacterial diarrhea is the most common, caused by pathogens such as *Escherichia coli*, *Salmonella*, and *Streptococcus* [2,3,4]. These pathogens spread via contaminated water, food, or environments. Lamb diarrhea not only directly affects growth and development but also exacerbates intestinal damage through inflammation, oxidative stress, intestinal epithelial barrier disruption, and intestinal microbiota imbalance. These pathological processes often form a vicious cycle, worsening the clinical symptoms of diarrhea. Studies have shown that diarrhea induces the release of intestinal inflammatory factors, such as IL-1β, IL-6, and TNF-α, which aggravate damage to the intestinal mucosa [5,6]. Meanwhile, oxidative stress activates inflammatory signaling pathways, further promoting intestinal injury, and imbalances in the intestinal epithelial barrier and intestinal microbiota increase the host’s susceptibility to pathogens [7,8,9,10]. Current treatments have limitations. For example, the use of antibiotics can lead to the emergence of drug-resistant pathogens and potential negative impacts on microbial diversity. These challenges underscore the urgent need for alternative strategies to prevent and treat lamb diarrhea.

The intestinal microbiota plays a key role in regulating metabolism, immune function, nutrient absorption, and maintaining the integrity of the intestinal barrier [11,12,13]. In recent years, probiotics have gained widespread attention for their potential to improve intestinal health, prevent diseases, and enhance production performance in animals. *Bacteroides fragilis*, a Gram-negative anaerobic bacterium, is widely found in the intestines of mammals. As an essential component of healthy microbiota, it is considered to have significant effects on the host’s immune regulation and anti-inflammatory response. Studies have shown that *Bacteroides fragilis* can promote the generation of regulatory T cells by producing metabolites such as polysaccharide A, thereby alleviating intestinal inflammation [14,15,16]. Furthermore, its unique metabolic characteristics enable it to degrade complex polysaccharides, improving the host’s energy utilization efficiency [17,18,19]. However, research on sheep-derived *Bacteroides fragilis* is limited, and its probiotic potential and effects on other animals have not been systematically explored.

This study hypothesizes that a sheep-derived *Bacteroides fragilis* isolate possesses significant probiotic potential, capable of alleviating intestinal inflammation, restoring intestinal microbiota homeostasis, and improving intestinal barrier function in lambs and mice. To test this hypothesis, we analyzed the growth characteristics of the *Bacteroides fragilis* isolate and evaluated its effects on intestinal microbiota, the inflammatory response, and growth performance through animal experiments. The findings aim to provide scientific evidence for the application of *Bacteroides fragilis* as a next-generation probiotic in animal husbandry.

## 2. Materials and Methods

### 2.1. Bacteroides fragilis

The *Bacteroides fragilis* isolate was isolated from the intestines of sheep by the microbiology department of the college of veterinary medicine, Yangzhou University [20].

### 2.2. Growth Characteristics of Bacteroides fragilis

#### 2.2.1. Acid and Bile Salt Tolerance

To evaluate the tolerance of *Bacteroides fragilis* to acidic and bile salt conditions simulating gastrointestinal transit, the pH of the culture medium was adjusted to 2.0, 3.0, 4.0, 5.0, 6.0, 7.0, and 8.0 using 1 mol/L hydrochloric acid. The culture was inoculated at 4% (*v*/*v*) into Gifu Anaerobic Medium (GAM) with these different pH values, cultured anaerobically at 37 °C for five hours, and the OD_600nm_ value was measured. The change in the OD_600nm_ value was calculated. Bovine bile salt was added to the culture medium to achieve concentrations of 0% (control group), 0.1%, 0.2%, and 0.3%. The culture was inoculated at 4% (*v*/*v*) into GAM medium with these different bile salt concentrations and cultured anaerobically at 37 °C for five hours. The number of viable bacteria was detected using the plate counting method.

#### 2.2.2. Simulated Artificial Gastric Fluid and Artificial Intestinal Fluid Tolerance Test

Artificial gastric fluid and artificial intestinal fluid were purchased as pre-prepared solutions from Beijing Baiao Leibo Technology Co., Ltd. (Beijing, China). These solutions were used directly according to the manufacturer’s instructions. The isolate was inoculated into GAM medium and cultured anaerobically at 37 °C for 24 h with shaking at 1000 rpm. After centrifugation at 5000 rpm for five minutes, the bacterial cells were collected and washed with PBS (phosphate-buffered solution) to prepare a suspension of 10^9^ CFU/mL. A total of 4.5 mL of artificial gastric fluid was adjusted to pH 3.0, filtered through a 0.22 μm filter, and reserved. A total of 0.5 mL of the bacterial suspension was added to 4.5 mL of artificial gastric fluid and artificial intestinal fluid, with three replicate groups. The mixture was cultured anaerobically at 37 °C with shaking for three hours. The number of viable bacteria was detected using the plate counting method.

#### 2.2.3. Antibiotic Sensitivity Test

The sensitivity of *Bacteroides fragilis* to common antibiotics (such as ampicillin, gentamicin, ciprofloxacin, etc.) was tested using the Kirby–Bauer disk diffusion method. The diameter of the inhibition zone was measured, and sensitivity was evaluated according to Clinical and Laboratory Standards Institute (CLSI) standards.

### 2.3. Effect of Bacteroides fragilis on Enterohemorrhagic Escherichia coli (EHEC)-Infected Mice

#### 2.3.1. Animal Grouping and Treatment

Fifty six-week-old male mice (BALB/c) were selected from the experimental animal center of Yangzhou University. Mice were not premedicated. After a three-day feeding period, the mice were divided into the following five groups, with 10 mice in each group: the Healthy Control group (CG), *Bacteroides fragilis* Treatment Group (NTBF), EHEC Infection Group (EHEC), EHEC + Low Dose *Bacteroides fragilis* Group (EHEC + LNTBF), and EHEC + High Dose *Bacteroides fragilis* Group (EHEC + HNTBF). The mice in the last three groups were gavaged with 100 μL of EHEC bacterial suspension (concentration adjusted to 10^6^ CFU/mL) on day 1, day 3, and day 5. After 24 h, the EHEC + LNTBF Group was gavaged with 200 μL of PBS and 200 μL of 10^9^ CFU/mL *Bacteroides fragilis* suspension, and the EHEC + HNTBF Group was gavaged with 200 μL of PBS and 200 μL of 10^10^ CFU/mL *Bacteroides fragilis* suspension. The NTBF group was gavaged with 200 μL of 10^9^ CFU/mL *Bacteroides fragilis* suspension, and the CG group was gavaged with PBS. The body weight of the mice was measured every two days starting from the first day of the experiment.

#### 2.3.2. Sample Collection

After one and two weeks, the mice were euthanized by cervical dislocation. The colon was dissected, with some parts stored at −70 °C and other parts preserved in 4% formaldehyde for further use.

#### 2.3.3. Histopathological Analysis

The tissues collected from the colon were subjected to routine paraffin embedding and sectioning, followed by Hematoxylin and Eosin (HE) staining. For each sample, five tissue sections were prepared and analyzed. The histopathological changes in the colon were scored. Histopathological changes included epithelial defects, edema, and lymphocyte, monocyte, plasma cell, neutrophil, and eosinophil infiltration. The scoring criteria were as follows (Table 1).

#### 2.3.4. Measurement of Inflammatory Factors and Oxidative Stress Indicators 

Frozen tissue samples were ground into powder using a precooled, sterilized liquid nitrogen mortar and pestle. One milliliter of IP lysis buffer was added to the tissue powder, and the mixture was homogenized. The homogenate was transferred into a centrifuge tube, sonicated at 200 W for five seconds, and centrifuged at 12,000 rpm for 30 min at 4 °C. The supernatant was collected for ELISA assays to measure the levels of IL-6 and TNF-α. The ELISA method was as follows: Each well received 100 μL of the sample and was cultured at 37 °C for 90 min. Then, 100 μL of biotin-labeled antibody was added per well and cultured at 37 °C for 60 min. The wells were washed three times with washing buffer, followed by the addition of 100 μL of ABC solution per well, and cultured at 37 °C for 30 min. After washing the wells five times, 90 μL of the substrate solution was added to each well and cultured at 37 °C in the dark for 25 min. After, 100 μL of stop solution was added per well, and the OD_450nm_ was recorded. The levels of IL-6 and TNF-α in the samples were determined using the standard curve. The MDA (malondialdehyde) and SOD (superoxide dismutase) levels, which are markers of oxidative stress, were measured using MDA and SOD kits (Nanjing Jiancheng Bioengineering Institute, Nanjing, China).

#### 2.3.5. Gene Expression Analysis

Total RNA was extracted from colon tissue using TRIzol reagent and reverse-transcribed, and the gene expression of tight junction proteins was analyzed using quantitative real-time PCR (qPCR). The relative mRNA expression levels of target genes (ZO-1 and Occludin) were calculated using the 2^−ΔΔCt^ method, with GAPDH serving as the internal reference gene. The ΔCt was obtained by subtracting the Ct value of GAPDH from that of the target gene, and the ΔΔCt was determined by normalizing the ΔCt values of each sample to the average ΔCt of the control group. The primers used are as follows (Table 2) [21]. The cycling conditions were initial denaturation at 95 °C for 10 min, 40 cycles at 95 °C for 15 s, 60 °C for 30 s, and 72 °C for 20 s.

### 2.4. Probiotic Effects of Bacteroides fragilis on Lambs

#### 2.4.1. Animal Grouping and Treatment

Approximately 10 healthy lambs (around three months old) and five diarrheic lambs were randomly selected from a sheep farm in Haimen District, Nantong City, Jiangsu Province. The selection criteria included the following. Healthy Lambs: Displaying no clinical signs of disease, with normal appetites, activity levels, and fecal consistency. Diarrheic Lambs: Identified based on the presence of loose or watery stools for at least two consecutive days, accompanied by reduced activity or appetite. All lambs were approximately three months old, of similar weight (10–15 kg), and were housed under consistent husbandry conditions, including unrestricted access to clean water and feed consisting of a standard farm diet. They were maintained in a controlled environment with proper ventilation, bedding, and routine farm management practices.

Lambs were randomly assigned into three groups. These were the Healthy Control Group (H): five healthy lambs, no gavage administration, the Healthy + *Bacteroides fragilis* Group (B): five healthy lambs, gavaged with *Bacteroides fragilis*, and the Diarrhea Group (F): five diarrheic lambs, gavaged with *Bacteroides fragilis*. The *Bacteroides fragilis* suspension was prepared at a concentration of 10^9^ CFU/mL. On day 0, rectal swab samples were collected for baseline microbiota analysis. Then, the lambs in both the Healthy + *Bacteroides fragilis* Group (B) and the Diarrhea Group (F) were gavaged with 5 mL of the *Bacteroides fragilis* suspension per 10 kg of body weight for five consecutive days. The control group (H) received no treatment. The *Bacteroides fragilis* dosage was adjusted according to the weight of each lamb to ensure proper administration.

#### 2.4.2. Sample Collection

Due to the health status of the lambs in the healthy control group, we selected four lambs from each group to ensure high-quality samples and reliable data. Selection was based on ensuring representative individuals within each group, consistent with the inclusion criteria described in Section 2.4.1. The selection process was based on the following criteria: 1. Health Status: Only healthy lambs in the Healthy Control Group with no clinical signs of disease (as described in Section 2.4.1) were included in the selection for analysis. 2. Consistency Across Groups: The lambs were chosen to ensure that they met the inclusion criteria specified for each group, with balanced representation from the Healthy Control Group, the Healthy + *Bacteroides fragilis* Group, and the Diarrhea Group. 3. Non-Interference with Future Sample Collection: Once a lamb was selected for a group, it remained part of that group for the duration of the study, ensuring consistency in the sampling process. Fresh rectal swab samples were collected from four selected lambs on day 0, day 1, day 3, and day 5 using sterile cotton swabs (once a lamb was selected, subsequent rectal swab collection could not be changed). The samples were numbered as follows: H0, H1, H3, H5, B0, B1, B3, B5, F0, F1, F3, and F5.

#### 2.4.3. 16S rRNA Sequencing Analysis

DNA was extracted from the rectal swab samples using a DNA extraction kit (TianGen Biochemical Technology Co., Ltd., Beijing, China). The V3–V4 hypervariable region of the bacterial 16S rRNA gene was amplified using the universal primers 341F (5′-CCTAYGGGRBGCASCAG-3′) and 806R (5′-GGACTACNNGGGTATCTAAT-3′). After PCR amplification, the target bands were purified and recovered using the Universal DNA purification kit (TianGen Biochemical Technology Co., Ltd., Beijing, China). Library construction was performed using the NEBNext^®^ Ultra DNA Library Prep Kit (New England Biolabs, Inc., Beijing, China), followed by high-throughput sequencing on the Illumina platform. The sequencing data were analyzed using QIIME2 (2023.5) to evaluate the changes in intestinal microbial community structure and diversity among the different treatment groups.

### 2.5. Statistical Analysis

Statistical analysis was performed using IBM SPSS Statistics 26. One-way analysis of variance (ANOVA) was conducted to evaluate the effects of different treatments on the levels of inflammatory factors and oxidative stress indicators. Tukey’s post hoc test was applied for multiple comparisons to identify specific group differences (*p* < 0.05 was considered statistically significant).

## 3. Results

### 3.1. Characteristics of Bacteroides fragilis

#### 3.1.1. Acid and Bile Salt Tolerance and Artificial Intestinal Fluid and Artificial Gastric Fluid Cultivation

**Figure 1:**
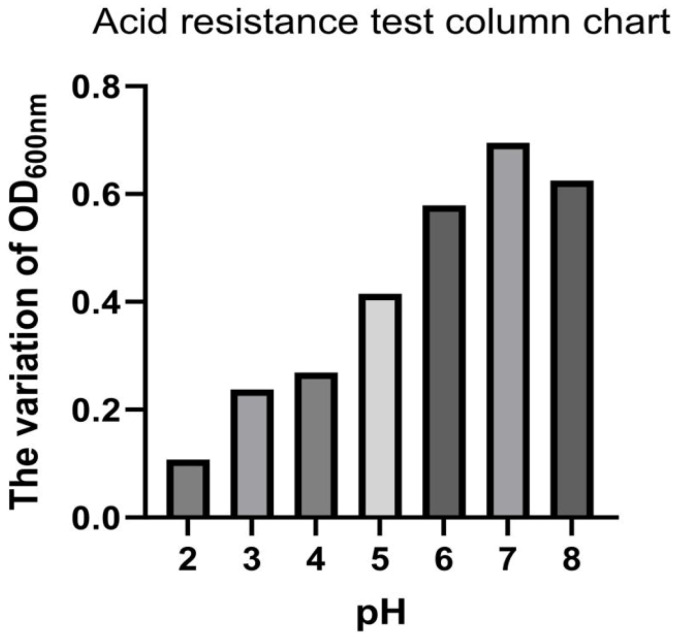
Changes in OD_600nm_ at different pH levels.

The results showed that *Bacteroides fragilis* can survive and grow under acidic conditions, with an optimal pH range of 6 to 8 (Figure 1). Growth varied under different bile salt concentrations, and the isolate demonstrated tolerance to artificial intestinal fluid and artificial gastric fluid (Table 3).

#### 3.1.2. Antibiotic Sensitivity

Antibiotic susceptibility tests indicated that *Bacteroides fragilis* was resistant to amoxicillin, compound trimethoprim, ciprofloxacin, cefotaxime, clarithromycin, tetracycline, ceftriaxone, doxycycline, norfloxacin, and amikacin, and it showed intermediate resistance to fluoroquinolone (Table 4).

### 3.2. Effects of Bacteroides fragilis on EHEC-Infected Mice

#### 3.2.1. Body Weight Changes

**Figure 2:**
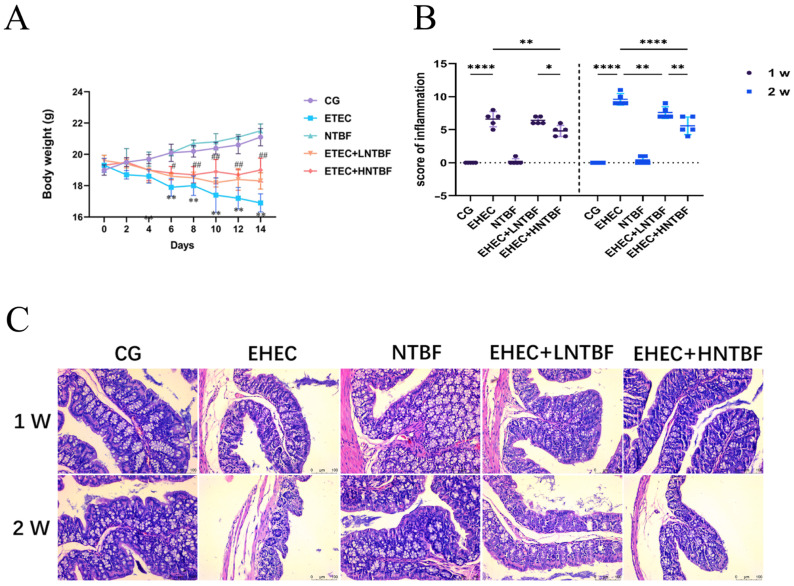
(**A**): Mice body weight changes; (**B**): Mice colon histopathological score; (**C**): Tissues from the colon. *: *p* < 0.05, **: *p* < 0.01, ****: *p* < 0.0001, #: *p* < 0.05, ##: *p* < 0.01.

The body weight of the mice in the CG and NTBF groups gradually increased with age, with the NTBF group surpassing the CG group in weight after one week, though the difference was not significant. The body weights of the EHEC group, the EHEC + LNTBF group, and the EHEC + HNTBF group decreased, with the degree of decline decreasing, respectively (Figure 2A).

#### 3.2.2. Histopathological Changes in the Intestine

Compared to the CG group, the EHEC group showed partial disappearance of the colonic villi, narrowing of the colonic crypts, and even structural damage, with irregular crypt surfaces and distorted villi along with inflammatory cell infiltration. The NTBF group showed no changes. In the EHEC + LNTBF group and the EHEC + HNTBF group, compared to the EHEC group, the structure of the tissue from the colon showed no improvement in the first week for the EHEC + LNTBF group, whereas the EHEC + HNTBF group showed a slight recovery. In the second week, both groups showed better recovery compared to the previous week. These results suggest that *Bacteroides fragilis* alone had no toxicity, but it promoted recovery in mice with inflammatory colonic damage (Figure 2B,C).

#### 3.2.3. IL-6 and TNF-α Levels

**Figure 3:**
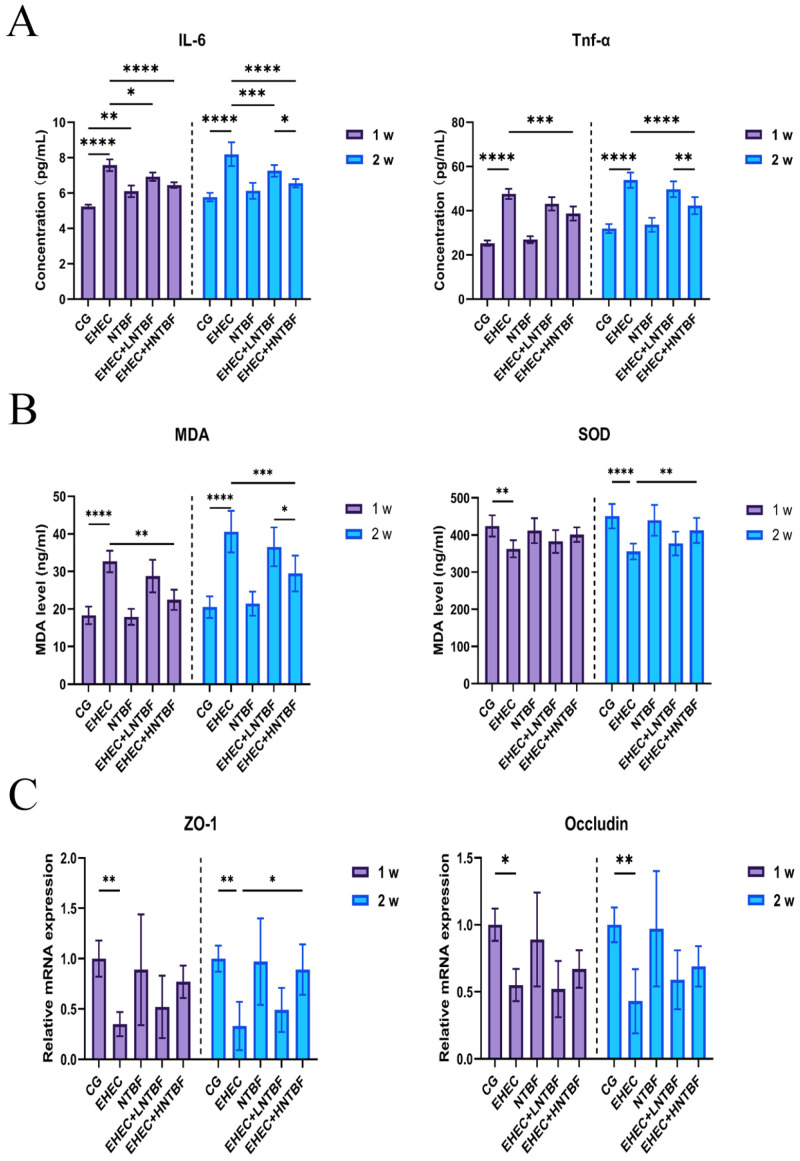
(**A**): Inflammatory factor levels in colon tissue; (**B**): Oxidative stress levels in colon tissue; (**C**): Barrier protein expression in colon tissue. *: *p* < 0.05, **: *p* < 0.01, ***: *p* < 0.001, ****: *p* < 0.0001.

At week 1, the EHEC group significantly increased the protein expression levels of IL-6 compared to the control group (*p* < 0.0001). In comparison to the CG group, the NTBF group showed significantly higher IL-6 protein expression levels (*p* < 0.01), while there was no significant difference in TNF-α levels between the NTBF and control groups (*p* > 0.05). The high-dose group significantly reduced IL-6 and TNF-α levels compared to the EHEC infection group (*p* < 0.0001 for IL-6 and *p* < 0.001 for TNF-α). At week 2, compared to the EHEC infection group, the high-dose group showed significant reductions in IL-6 and TNF-α expression (*p* < 0.0001 for IL-6 and *p* < 0.001 for TNF-α). The low-dose group significantly reduced IL-6 levels (*p* < 0.001), but there was also a reduction in TNF-α levels, with significant differences between the EHEC + HNTBF group and the EHEC + LNTBF group for TNF-α expression (*p* < 0.01) (Figure 3A).

#### 3.2.4. Oxidative Stress Markers

At week 1, the EHEC group significantly increased MDA levels compared to the CG group (*p* < 0.0001) and significantly reduced SOD levels (*p* < 0.01). The EHEC + HNTBF group significantly lowered MDA levels compared to the EHEC group (*p* < 0.01). At week 2, the EHEC group had significantly higher MDA levels (*p* < 0.0001) and significantly lower SOD levels (*p* < 0.0001) compared to the CG group. The EHEC + HNTBF group significantly reduced MDA levels (*p* < 0.001) and significantly increased SOD levels compared to the EHEC group (*p* < 0.01) (Figure 3B).

#### 3.2.5. ZO-1 and Occludin Expression

At week 1, the EHEC group showed significantly lower gene expression levels of ZO-1 (*p* < 0.01) and occludin (*p* < 0.05) compared to the CG group. In contrast, both the EHEC + LNTBF group and the EHEC + HNTBF group exhibited higher gene expression levels of ZO-1 and occludin compared to the EHEC group, with the EHEC + HNTBF group showing higher levels than the EHEC + LNTBF group. There were no significant differences between the NTBF group and the CG group. At week 2, the EHEC group still showed significantly lower expression levels of ZO-1 and occludin compared to the control group (*p* < 0.01 for ZO-1 and *p* < 0.05 for occludin). Both the EHEC + HNTBF group and the EHEC + LNTBF group had significantly higher ZO-1 gene expression than the EHEC group (*p* < 0.05), with no significant difference between the EHEC + HNTBF group and the EHEC + LNTBF group (*p* > 0.05) (Figure 3C).

### 3.3. Probiotic Effects of Bacteroides fragilis on Lambs

#### 3.3.1. Health Status of Lambs

In the diarrhea group, five lambs received *Bacteroides fragilis* culture orally. By day 3, four of them stopped having diarrhea. The remaining lamb, which did not recover, showed signs of progressive emaciation, with tail fur around the anus covered in feces, weakness in the legs, a sunken rib cage, and frequent lying down in the pen. No significant changes were observed in the other two groups.

#### 3.3.2. Analysis of Intestinal Microbiota Abundance 

**Figure 4:**
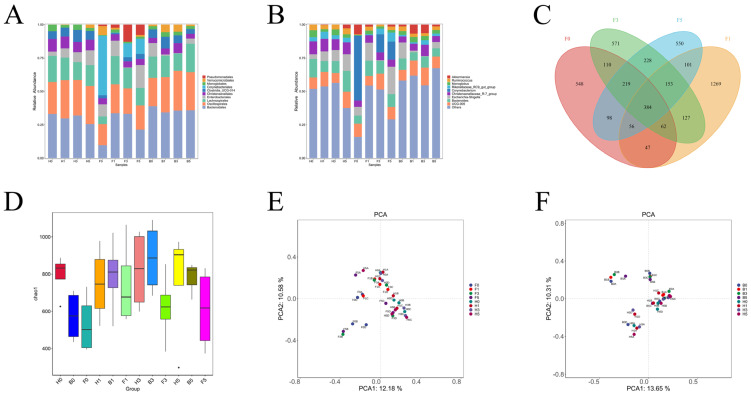
(**A**): Top 10 phylum-level microbial abundances in sheep intestines; (**B**): Top 10 genus-level microbial abundances in sheep intestines; (**C**): Venn diagram of the diarrheal group; (**D**): Chao1 index box plot for the diarrheal group; (**E**): PCA analysis for the diarrheal group; (**F**): PCA analysis for the healthy + *Bacteroides fragilis* group.

In the healthy control group, the intestinal microbiota composition remained stable over the 5-day period and was predominantly composed of Firmicutes and Bacteroidota, with Proteobacteria and Campylobacterota present in smaller amounts. The Firmicutes/Bacteroidota (Fir/Bac) ratio exhibited minor fluctuations, starting at 2.16 on day 0 and slightly decreasing to 2.06 by day 5. Conversely, in the diarrhea group, Actinobacteria initially accounted for 35.5% but sharply decreased after *Bacteroides fragilis* gavage, partially recovering to 10.75% by day 5. Similarly, the Fir/Bac ratio in this group dropped from 6.01 on day 0 to 2.32 on day 5. In the healthy + *Bacteroides fragilis* group, the Fir/Bac ratio increased slightly from 1.58 to 1.75, with a decrease in Proteobacteria from 14.27% to 3.67% (Figure 4A).

At the genus level, the healthy control group exhibited stable core microbiota, including *Christensenellaceae_R-7_group*, *Bacteroides*, and *UCG-005*, with minor variations in their relative abundances. The diarrhea group exhibited initial intestinal dysbacteriosis. On day 1, after *Bacteroides fragilis* gavage, the abundance of intestinal microbiota was similar to that of the healthy control group. From day 3 to day 5, there was a rebound in *Corynebacterium* and an increase in *Escherichia-Shigella*. In the healthy + *Bacteroides fragilis* group, *Akkermansia* abundance peaked at 5.27% on day 1 before declining to 1.09% by day 5 (Figure 4B). Additionally, the diarrhea group had 384 core species across the four time points. The number of species was 1140 on day 0, 1824 on day 1, 1470 on day 3, and 1405 on day 5. On day 0, prior to *Bacteroides fragilis* gavage, diarrhea caused a reduction in the number of species, but after 1 day of gavage, the number of species at the genus level increased dramatically. From day 3 to day 5, the number of species decreased, but it stabilized at a higher level than before *Bacteroides fragilis* gavage (Figure 4C).

The Chao1 index demonstrated stable species richness in the healthy control group, with slight fluctuations. In the diarrhea group, species richness increased after *Bacteroides fragilis* gavage on day 1 and declined from day 1 to day 5, although it was still higher than on day 0. The healthy + *Bacteroides fragilis* group showed a consistent increase in species richness, indicating a positive effect of *Bacteroides fragilis* gavage on microbiota diversity (Figure 4D).

Between the diarrhea group and the healthy group, PCA analysis showed significant differences in intestinal microbiota. The first principal component (PCA1) and the second principal component (PCA2) contributed 15.86% and 11.32%, respectively, in the diarrhea group. On day 1 after *Bacteroides fragilis* gavage, the microbiota composition in the F1 was similar to that in the H1, indicating a significant improvement in the microbiota due to *Bacteroides fragilis*. However, over time, recovery varied among individual lambs (Figure 4E). In the healthy + *Bacteroides fragilis* group, PCA analysis showed that PCA1 and PCA2 contributed 19.84% and 14.91%, respectively. On day 1, the microbiota in the B1 was similar to that in the H1, suggesting that *Bacteroides fragilis* improved the microbiota composition, though the effect decreased by day 3, with a growing difference between the B3 and H3 (Figure 4F).

## 4. Discussion

With the widespread application of probiotics in healthcare and food engineering, there has been increasing interest in isolating and studying new probiotic strains. *Bacteroides fragilis*, as a “second-generation probiotic”, stands out due to its antibiotic resistance [22] and remarkable probiotic effects, drawing attention from researchers. This study aimed to explore the growth characteristics and probiotic effects of a *Bacteroides fragilis* isolate derived from sheep, providing scientific evidence for its application in animal husbandry.

The growth characteristics of *Bacteroides fragilis* revealed that bacterial growth was affected at a pH of 5, but it exhibited strong resilience. At pH 3 and 4, the bacteria grew more slowly but remained active. At pH 2, growth was extremely slow, with nearly no growth. This suggests that the isolate can tolerate the acidic environment in the stomachs of ruminants. The concentration of bile salts in the intestines of livestock and poultry is approximately 0.03% to 0.3%. When exogenous bacteria enter the intestines, they are inhibited by bile salts. Therefore, to survive and grow in the intestines, bacteria must tolerate bile salts at concentrations up to 0.3%. In this study, the survival rate was higher at 0.1% bile salts, significantly affected at 0.2%, and lower at 0.3%. Regarding antibiotic resistance, *Bacteroides fragilis* demonstrates resistance to most antibiotics due to its multidrug efflux pump mechanism [22], consistent with this study. This indicates that the *Bacteroides fragilis* in sheep farming would not be significantly interfered with by antibiotics. Therefore, future research should focus on optimizing the strain’s acid and bile salt tolerance further to improve its survivability and colonization efficiency in vivo. These in vitro characteristics clearly showed that this strain has the potential for further development as a probiotic.

Infection with EHEC can cause inflammation, where the key virulence factor, the attaching and effacing gene (*eae*), leads to the adhesion and damage of intestinal epithelial cells [23]. EHEC binds to the host’s intestinal epithelial cells through tight junction proteins, causing cell damage and impairing the intestinal barrier. Damage to the intestinal barrier is a major cause of intestinal damage, with alterations in the expression of tight junction proteins such as claudin-1, occludin, and ZO-1. Studies have shown that viral or bacterial infections that lead to intestinal inflammation often result in significant reductions in the expression of these proteins, indicating the severity of intestinal damage. In this study, positive effects were observed in BALB/c mice, specifically the alleviation of EHEC-induced intestinal barrier damage, with the upregulation of occludin and ZO-1. This finding suggests that *Bacteroides fragilis* can help restore intestinal barrier integrity, which is a crucial factor in preventing infections and maintaining overall intestinal health. Both high-dose and low-dose *Bacteroides fragilis* showed therapeutic effects, and the high-dose group showed better efficacy than the low-dose group, indicating the need for further optimization of the optimal dosage for treatment. However, the effects observed in mouse models cannot fully replicate the outcomes in lambs. The gastrointestinal physiology, immune system, and microbiota composition of the two species differ significantly. Therefore, while data from mice provided valuable mechanistic insights, trials with lambs are critical for assessing the translational relevance of our findings in an agricultural context.

The results of 16S rRNA sequencing analysis indicate that *Aerococcus suis* and *Corynebacterium camporealensis* were found to be significantly overrepresented in the diarrheal group in lambs. Additionally, *Methanobrevibacter smithii* emerged as a highly abundant bacterium in the unrecovered lamb in the diarrheal group. *Aerococcus suis* is a less commonly isolated bacterium whose virulence and pathogenicity are not well-understood. It has been isolated from pigs and sheep [24,25] and is possibly linked to subclinical mastitis in sheep [26]. *Methanobrevibacter smithii*, which was highly abundant in the diarrheal group, has been associated with irritable bowel syndrome (IBS) and may play a role in the development of diarrhea [27,28]. The high abundance of these three bacteria in the intestinal microbiota suggests that they may be associated with diarrhea. The lamb trials show that the *Bacteroides fragilis* isolate has a clear impact on the intestinal microbiota of lambs suffering from diarrhea. There was a significant reduction in the abundance of potentially pathogenic bacteria (*Aerococcus suis* and *Corynebacterium camporealensis*) within one day of probiotic gavage. Probiotics exert their beneficial effects through various mechanisms, including lowering intestinal pH, reducing pathogen colonization and invasion, and modulating the host’s immune response [29]. These results suggest that *Bacteroides fragilis* may be contributing to a healthier intestinal environment, potentially through competitive exclusion or modulation of the host immune response. However, the therapeutic effects were not sustained in subsequent days, likely due to an insufficient dosage or a low colonization rate. A diverse microbiota is critical for maintaining gut homeostasis, resilience to pathogenic invasions, and optimal immune function. Reduced diversity is a hallmark of dysbiosis, often associated with diarrhea and other gastrointestinal disorders. Alpha diversity analysis showed that the species count in the diarrheal group significantly increased after *Bacteroides fragilis* treatment, similar to the healthy control group. This finding aligns with previous research, which reported that probiotics can treat small intestinal bacterial overgrowth [30]. By restoring microbial diversity to levels similar to healthy controls, *Bacteroides fragilis* helps re-establish a balanced microbial ecosystem, potentially enhancing gut barrier function, nutrient absorption, and overall health.

In addition, the ratio of Firmicutes to Bacteroidota (Fir/Bac) is commonly associated with intestinal microbiota in obese individuals [31] and has been shown to change with age in humans [32]. In this study, the average Fir/Bac ratio in the healthy control group was 2.19. In the diarrheal group, the Fir/Bac ratio was significantly higher on day 0 (6.01) and decreased to 2.32 by day 5 after probiotic gavage. This suggests that the probiotic treatment improved the intestinal microbiota composition, bringing the ratio closer to that of the healthy control group. The health probiotic group also showed a slight increase in the Fir/Bac ratio, further indicating an improvement in intestinal health. Similar findings have been reported in studies where probiotics restored the Fir/Bac ratio in various diseases, including chronic stress- and obesity-induced dysbiosis [33,34]. Thus, these results show the potential of using Fir/Bac ratios as an indicator of intestinal health and treatment efficacy.

In this study, the sample size in our lamb trial was relatively small, and key parameters, such as weight changes and feed intake, were not measured. Moreover, comprehensive analyses, including metagenomics and metabolomics, are needed to better understand the mechanisms through which probiotics exert their effects and their potential impact on the existing intestinal microbiota. Additionally, the lack of long-term follow-up data prevents us from assessing the sustainability of the probiotic effects and the long-term benefits for lamb health. The administration of the probiotic through gavage might not be the most efficient method and might not be reproducible in real farming conditions. Future studies should focus on optimizing delivery methods and assessing the long-term impacts on the host, including growth rates and overall animal welfare. The assessment of different doses might also help improve the efficacy of the probiotic.

## 5. Conclusions

This study showed that sheep-derived *Bacteroides fragilis* exhibits strong acid and bile salt tolerance, antibiotic resistance, and significant probiotic potential, particularly in restoring intestinal barrier integrity and improving the composition of intestinal microbiota. Although *Bacteroides fragilis* showed positive effects in reducing pathogenic bacteria, the effects were not sustained, highlighting the need for optimizing dosage, delivery methods, and colonization efficiency. Future research should focus on long-term trials, incorporating advanced analytical approaches to better understand the practical applications of *Bacteroides fragilis* in animal husbandry.

## Figures and Tables

**Table 1:** Colonic pathology score.

**Item**	**Score**			
	**0**	**1**	**2**	**3**
Epithelial defects	None	Mild	Moderate	Severe
Edema	None	Mild	Moderate	Severe
Lymphocyte, monocyte, and plasma cell infiltration	None	Mild	Moderate	Severe
Neutrophil infiltration	None	Mild	Moderate	Severe
Eosinophil infiltration	None	Mild	Moderate	Severe

**Table 2:** Primers for qPCR.

Gene	Primers
ZO-1	F: CGGGCTACCTTATTGAATGTCC
	R: GAGCGAACTGAATGGTCTGATG
Occludin	F: GGATGACTACAGAGAGGAGAGGG
	R: CATAGTCTCCCACCATCCTCTTG

**Table 3:** Survival Rate of *Bacteroides fragilis*.

Solution	Survival Rate (%)
Artificial intestinal fluid	92.22
Artificial gastric fluid	38.89
0.1% bile salt	82.31
0.2% bile salt	42.42
0.3% bile salt	13.92

**Table 4:** Sensitivity of *Bacteroides fragilis* to Different Antibiotics.

Antibiotic	Inhibition Zone (mm)	Evaluation
Amoxicillin	0	R
Florfenicol	13	I
Compound Sulfamethoxazole Tablets	0	R
Ciprofloxacin	0	R
Cefotaxime	0	R
Clarithromycin	0	R
Tetracycline	6	R
Doxycycline	10	R
Ceftriaxone	0	R
Norfloxacin	0	R
Amikacin	0	R

## Data Availability

The raw data supporting the conclusions of this article will be made available by the authors on request.
